# Primary hepatic lymphoma of MALT type mimicking hepatic adenoma treated by hepatectomy: a case report and literature review

**DOI:** 10.3389/fsurg.2023.1169455

**Published:** 2023-05-12

**Authors:** Ren-long Wang, Jia Wang, Yong-sheng Li, Yuan Wang, Qiong Su

**Affiliations:** ^1^ Department of General Surgery, Fifth School of Medicine/Suizhou Central Hospital, Hubei University of Medicine, Suizhou, China; ^2^ Department of Radiology, Fifth School of Medicine/Suizhou Central Hospital, Hubei University of Medicine, Suizhou, China; ^3^ Department of Pathology, Fifth School of Medicine/Suizhou Central Hospital, Hubei University of Medicine, Suizhou, China; ^4^ Department of Respiratory Medicine, Fifth School of Medicine/Suizhou Central Hospital, Hubei University of Medicine, Suizhou, China

**Keywords:** primary hepatic lymphoma, mucosal-associated lymphoid tissue, non-Hodgkin lymphoma, hepatectomy, case report

## Abstract

**Background:**

Primary hepatic lymphoma (PHL) is a rare malignant tumor. Extranodal marginal zone lymphoma of the mucosa-associated lymphoid tissue (MALT) is an indolent lymphoma occurring at extranodal sites. The stomach is the most common organ affected by MALT lymphoma, whereas liver-related lymphoma is rarely reported. Its atypical clinical presentation often delays the diagnosis. Owing to the rarity of PHL, identifying its optimal treatment still remains a challenge. Herein, we report a case of PHL of the MALT type mimicking hepatic adenoma that was treated by hepatectomy without chemotherapy and review the scarce literature. Our findings suggest that surgery is an alternative approach to cure patients with localized hepatic lymphoma.

**Case summary:**

A 55-year-old woman was admitted to our hospital because of upper abdominal discomfort, and a liver lesion was detected by computed tomography. She did not have nausea, fever, fatigue, jaundice, weakness, night sweats, or weight loss before admission. And her previous medical history was unremarkable. There were no positive signs on physical examination. Based on her preoperative examination including magnetic resonance imaging, the liver lesion was suspected to be a hepatic adenoma; however, the possibility of it being a malignancy like hepatocellular carcinoma was not excluded. Therefore, a decision of resection of the lesion was made. During the operation, hepatectomy of segment 4b and cholecystectomy were performed. The patient recovered well; however, after postoperative pathological examination, the lesion was diagnosed as a hepatic lymphoma of MALT type. The patient was reluctant to undergo chemotherapy or radiotherapy. At 18-month follow-up, no significant recurrence was observed, indicating that the treatment had a curative effect.

**Conclusion:**

Notably, primary hepatic lymphoma of MALT type is a rare, low-grade B-cell malignancy. Making an accurate preoperative diagnosis of this disease is usually difficult, and liver biopsy is an appropriate avenue to improve the diagnostic accuracy. In patients with a localized tumor lesion, hepatectomy followed by chemotherapy or radiotherapy should be considered to achieve better outcomes. Although this study describes an unusual type of hepatic lymphoma mimicking a benign tumor, it has its inherent limitations. More clinical studies are required to establish guidelines for the diagnosis and treatment of this rare disease.

## Introduction

Primary hepatic lymphoma (PHL) or liver lymphoma is a lymphoproliferative disorder that typically presents as a lesion confined to the liver without the involvement of lymph nodes, spleen, bone marrow, or other lymphoid structures. Diffuse large B-cell lymphoma (DLBCL), follicular lymphoma, T-cell lymphoma, and mucosal-associated lymphoid tissue (MALT) lymphoma constitute the majority of primary hepatic lymphomas. PHLs account for 0.016% of all cases of non-Hodgkin lymphoma (NHL) (1), and a primary hepatic extranodal marginal zone lymphoma of MALT is extremely rare. The etiology of PHL remains unclear, and due to its nonspecific clinical, laboratory, and imaging findings, the preoperative diagnosis is more difficult and usually inaccurate (2). Owing to its rarity, our current understanding of PHL is predominantly derived from case reports and case series (3). Consequently, to our knowledge, there is currently no consensus on imaging study findings or treatment for PHL. Herein, we present an interesting case of a primary hepatic lymphoma of MALT type treated with liver resection, which was preoperatively identified to be a hepatic adenoma.

## Case presentation

A 55-year-old woman consulted our hospital for investigation of upper abdominal discomfort, and then, a liver lesion was detected by computed tomography (CT) scan in April 2021. She did not have nausea, fever, fatigue, jaundice, weakness, night sweats, or weight loss before admission. The patient was previously healthy and had no history of hypertension, diabetes, heart disease, surgery, or allergies. Also, she had no remarkable personal history and no special family history. Physical examination at the time of admission to the hospital revealed no tenderness or rebound pain in the abdomen; furthermore, no lymphadenectasis, hepatomegaly, splenomegaly, or palpable mass was noted.

Laboratory data are shown in Table 1. Peripheral blood cell counts revealed decreased white cell count (2.8 × 10^9^/L, normal range 3.5%–9.5 × 10^9^/L) without anemia. Liver function and renal function were normal, and blood glucose, electrolyte, and lactate dehydrogenase (LDH) levels were in the normal range. Total cholesterol was slightly elevated (5.54 mmol/L, normal range 2.8–5.2 mmol/L). Serological tests for hepatitis B and C viruses (HBV and HCV) and human immunodeficiency virus (HIV) were all negative. Levels of tumor markers, such as alpha-fetoprotein (AFP), carcinoembryonic antigen (CEA), and carbohydrate antigen 19–9 (CA19-9), were within the normal range at 2.59 ng/ml, 1.01 ng/ml, and 5.62 U/ml, respectively.

**Table 1:** Relevant laboratory analysis.

Laboratory parameter	Value/unit	Reference range
Complete blood count Hemoglobin	126 g/L	115–150
Median globulin volume	93.5 fl	82.0–100.0
Median hemoglobin volume	29.0 pg	27.0–34.0
Leucocytes	2.8 × 10^9^/L	3.5–9.5
Neutrophils	1.27 × 10^9^/L	1.80–6.30
Lymphocytes	1.13 × 10^9^/L	1.10–3.20
Eosinophils	0.11 × 10^9^/L	0.02–0.52
Basophils	0.08 × 10^9^/L	0–0.06
Platelet count	216 × 10^9^/L	125–350
Coagulation INR	0.95	0.87–1.39
Biochemistry Aspartate aminotransferase	12 U/L	13–35
Alanine aminotransferase	9 U/L	7–40
Gamma-glutamyl transferase	12 U/L	7–45
Alkaline phosphatase	45 U/L	50–135
Lactate dehydrogenase	161.0 U/L	120.0–250.0
Total bilirubin	12.1 µmol/L	0–17.1
Total albumin	39.5 g/L	40.0–55.0
Glucose	4.52 mmol/L	3.89–6.11
Urea nitrogen	6.14 mmol/L	2.60–7.50
Creatinine	57.2 µmol/L	41.0–73.0
Sodium	141 mmol/L	137–147
Potassium	3.70 mmol/L	3.50–5.30
Chloride	108 mmol/L	99–110
Calcium	2.32 mmol/L	2.11–2.52
Total cholesterol	5.54 mmol/L	2.80–5.20
Triglyceride	1.19 mmol/L	0.38–1.70
Tumor markers Alpha-fetoprotein	2.59 ng/ml	0–10.0
Carcinoembryonic antigen	1.01 ng/ml	0–5.00
Carbohydrate antigen 19–9	5.62 U/ml	0–35.0
Carbohydrate antigen 15–3	4.35 U/ml	0–35.0
Carbohydrate antigen 125	8.06 U/ml	0–35.0
Carbohydrate antigen 724	2.19 IU/ml	0–6.00

Major changes are shown in bold.

**Figure 1:**
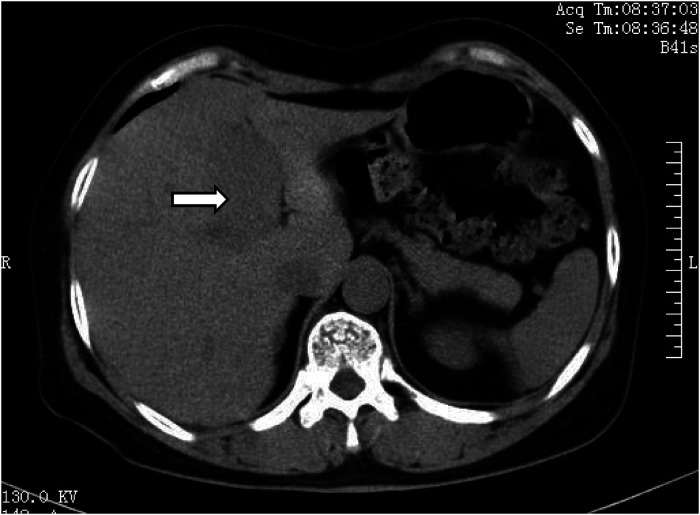
Abdominal computed tomography showing an elliptical low-density mass (white arrow) in the left inner lobe of the liver.

**Figure 2:**
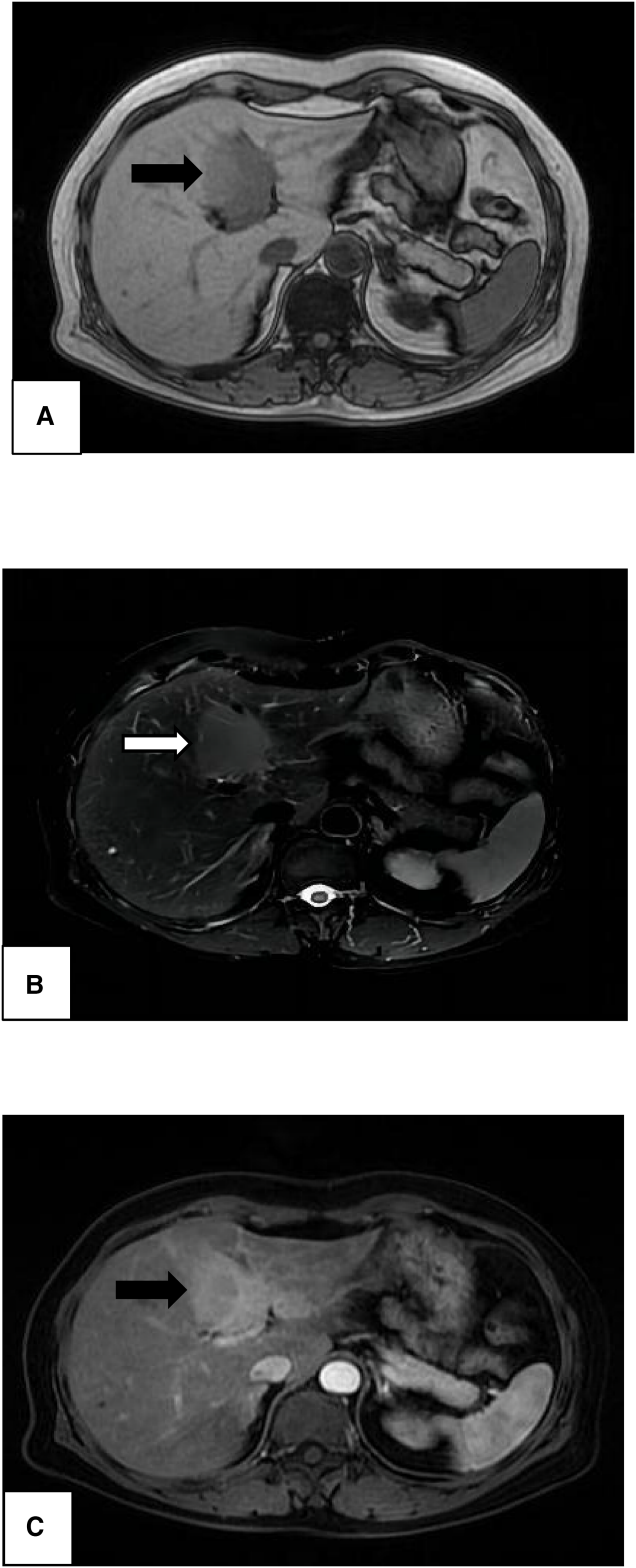
Magnetic resonance imaging of the liver showing a high-signal-intensity mass in both T1-weighted image (**A**, black arrow) and T2-weighted image (**B**, white arrow). Significant enhancement (**C**, black arrow) was noted in the enhanced arterial phase, revealing a mass of 4.7 × 4.2 cm in size.

Abdominal CT revealed an elliptic low-density shadow (Figure 1) occupying the left inner lobe of the liver. Then, abdominal magnetic resonance imaging (MRI) was performed, which showed a slightly longer signal with heterogeneous density in both T1 and T2 sequences. The mass measured 4.7 × 4.2 cm (Figure 2), after intravenous injection of contrast agent, and the lesion was significantly strengthened in the arterial stage and decreased in the portal and delayed stages, which was similar to the liver parenchyma enhancement. In addition, no neoplasm was found in subsequent CT scans of the cephalothorax, abdomen, and pelvic cavity.

According to the laboratory examination and imaging results, the liver lesion was suspected to be a hepatic adenoma, but the possibility of a malignancy like hepatocellular carcinoma was not excluded. The patient did not consent to a liver biopsy because of concerns about needle tract metastasis. The tumor was likely to be resectable, therefore, an exploratory surgery was performed three days after admission. During the surgery, a solid and well-circumscribed mass was found on the lobus quadratus adjacent to the portal fissure, and then, hepatectomy of segment 4b and cholecystectomy were performed. There were no palpable lymph nodes in the hepatoduodenal ligament or mesentery. The gastrointestinal tract was also normal. After the operation, the patient was treated by fasting prohibition, acid inhibition, and nutritional support, but she refused to undergo subsequent chemotherapy or radiotherapy. She had an uneventful recovery without postoperative bleeding or bile leakage, and the standardized classification of surgical complications (Clavien–Dindo) was stage I.

**Figure 3:**
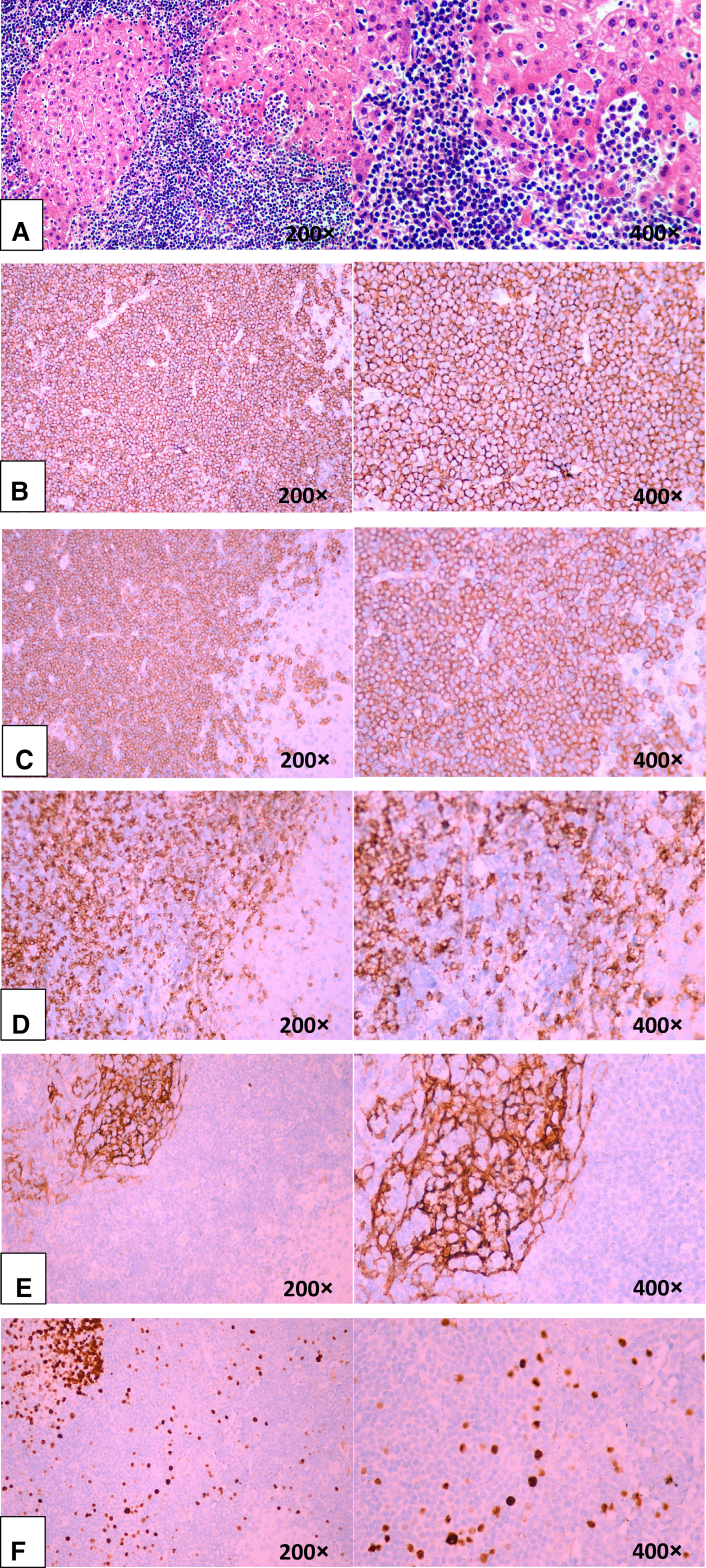
Microscopic findings of the liver confirming B-cell non-Hodgkin's lymphoma of MALT type. (**A, B, C, D, E,** and **F**). (**A**) Medium-sized lymphoid cells infiltrated into the liver epithelium, forming lymphoepithelial lesions (hematoxylin-eosin staining). (**B**) Positive for the B-cell marker CD20. (**C**) Positive for the B-cell marker CD79α. (**D**) Negative for CD5. (**E**). CD21 showed irregular follicular dendritic cell networks. (**F**) A relatively low proliferation fraction detected by Ki67 immunostaining (∼13%).

Unexpectedly, a hepatic lymphoma was diagnosed by postoperative histopathological examination, which showed medium-sized lymphocyte infiltration in the hepatic sinuses (Figure 3A). These heterotypic lymphocytes morphologically resemble central cells, which are arranged concentrically around reactive lymphoid follicles. Immunohistochemistry was positive for the pan-B-cell marker CD20 and for CD79α, CD43, and Bcl-2; conversely, it was negative for CD3, CD5, cyclinD1 (Figures 3B–D). Moreover, CD21 shows irregular follicular dendritic cell networks (Figure 3E). The lymphoid cells showed a relatively low proliferation fraction as detected by Ki67 immunostaining (Ki-67 proliferation index: ∼13%, Figure 3F). All these histologic findings confirmed B-cell non-Hodgkin's lymphoma of MALT type.

**Figure 4:**
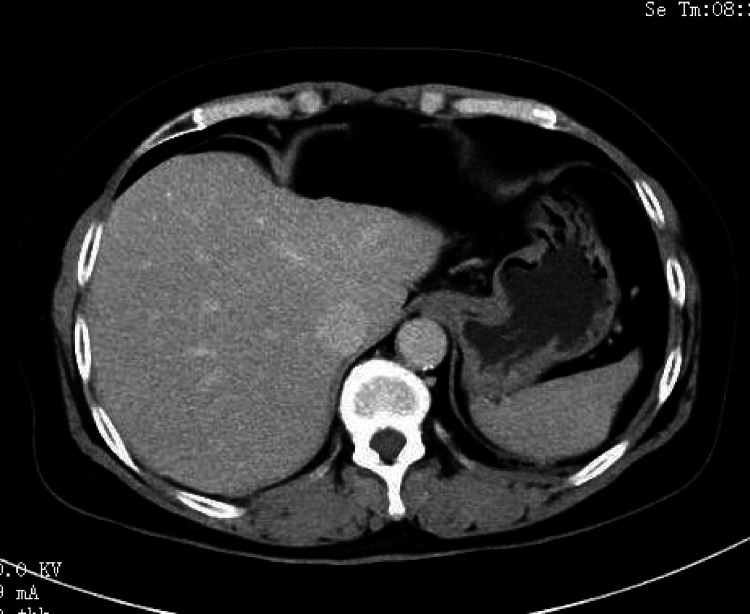
The recent systemic CT scan revealed no recurrence.

After discharge, this patient was referred to the department of hematology for follow-up. However, as the patient was reluctant to undergo chemotherapy or radiotherapy, she was reviewed approximately every six months without special intervention or medication. Fortunately, she was disease-free 18 months after the operation and the recent systemic CT scan revealed no recurrence (Figure 4).

## Discussion

Primary hepatic lymphoma is a lymphoid tissue proliferative disease with no extrahepatic infiltration (4). PHLs account for 0.1% of all liver malignancies, 0.4% of extranodal lymphomas, and 0.016% of all NHLs (5). In previous case studies, primary hepatic lymphoma that is predominantly of B-cell origin was found to account for the majority. Marginal zone lymphomas of MALT type are a group of B-cell neoplasms that involve extranodal tissues and usually have indolent clinical behavior (6). These lymphomas may arise in different anatomic locations, with the stomach being the most common site. This disease can affect anyone, but it most commonly occurs in people in their fifth or sixth decade, with a sex ratio of 2–3 men to one woman (7).

The etiology of PHL occurrence is still unclear. Currently, there are two vigorous controversies: infection and compromised immunity. Recently, several studies have suggested that the increased incidence of PHL could be attributed to systemic lupus erythematosus, liver cirrhosis, immunosuppressive therapy, and virus infections, such as Epstein–Barr virus (EBV), HIV, HCV, and human *T*-cell lymphotropic virus (8–10). For instance, Imrani et al. (11) reported a case of PHL in a patient with liver cirrhosis caused by hepatitis C infection. Furthermore, PHL was commonly noted in organ transplant recipients (12, 13). From these findings, it is clear that the immune environment plays an important role in the development and progression of PHL. However, it is important to note that the patient in this case did not have any of the above risk factors or conditions.

A patient with PHL may have nonspecific symptoms, such as right upper quadrant or epigastric pain, nausea, jaundice, fatigue, fever, or weight loss. In most cases, hepatomegaly is present. Lab tests typically indicate normal liver function, high levels of alkaline phosphatase and LDH, and normal levels of tumor markers, such as AFP and CEA (9). Tumor markers can contribute to the differential diagnosis of hepatocellular carcinoma or metastatic disease. In our case, all laboratory findings, including those of the complete blood count and liver function tests, were normal, and tumor markers were negative.

Imaging manifestations of hepatic lymphoma are extremely diverse and nonspecific, and a solitary hypoattenuating lesion with a core area of low intensity, which is indicative of necrosis, is the most typical presentation on CT (14, 15). In addition, MRI findings vary, with most lesions appearing hypointense on T1-weighted images and hyperintense on T2-weighted images (9), which was the case with our patient as well. Owing to its property of being taken up by the hepatocytes in later phases, gadolinium ethoxybenzyl diethylenetriamine pentaacetic acid (Gd-EOB-DTPA) has recently come to be used to find the malignant hepatic lesions. Albano et al. (16) demonstrated that mantle cell lymphoma (MCL) is an FDG-avid lymphoma and that 18F-FDG-PET/CT is a useful diagnostic tool.

Hepatic adenoma is a rare benign solid tumor with a 1:100,000 incidence that usually affects women in reproductive age and is solitary in 80% of cases. It has a soft, well-defined consistency with little or no fibrous capsule. Clinically, it is typically asymptomatic. It may present with elevated gamma glutamyl transferase and alkaline phosphatase (17). It commonly appears isointense to slightly hyperintense on T1- and T2-weighted imaging and shows moderate enhancement in the arterial phase with no persistent enhancement on the portal venous and delayed phases (18).

Due to its nonspecific clinical presentation and laboratory and radiologic features, PHL may be misdiagnosed as diseases that exhibit hyperintense signals on MRI, including primary hepatocellular carcinoma, hepatic adenoma, hepatitis, liver abscess, or metastatic liver disease. We searched the PubMed and Scopus databases for relevant English literature (including small series and single case reports) on the misdiagnosis of primary hepatic lymphoma in the last 20 years. Our key search terms used included “lymphoma,” “non-Hodgkin lymphoma,” “B-cell lymphoma,” “*T*-cell lymphoma,” “mucosal-associated lymphoid tissue,” “hepatocellular carcinoma,” “hepatitis,” “hepatic adenoma,” “liver abscess,” and “hepatic metastasis,” among others. In total, 14 cases with misdiagnosis were identified and reviewed (19–31). Case characteristics, such as sex and age of patients, diagnostic modality, and invasive measure, were evaluated and analyzed (Table 2). Six of them were misdiagnosed as HCC, four as hepatitis, two as metastases, and one each as hepatic cyst and cholangiocarcinoma. In our case, we considered it to be a hepatic adenoma before the operation on the basis of the similar MRI manifestation. A liver biopsy is needed to establish a definitive diagnosis of PHL. However, liver biopsy was not performed in our case because there were no laboratory findings that suggested PHL; the patient did not consent to a liver biopsy because of concerns about needle tract metastasis; the lesion was localized in the left lobe of the liver, and it was considered and proved to be resectable.

**Table 2:** Reported cases of primary hepatic lymphoma of MALT type misdiagnosed as other disease.

Author, year	Gender, age	Pathology type	Diagnostic modality	Diagnostic hypothesis	Invasive measure
Kaneko 2011 (19)	M	DLBL	CT/MRI	HCC	Liver biopsy
Bohlok 2018 (20)	M,68	MALT	FDG PET-CT	HCC	Surgery
Lee 2017 (21)	M,50	PTCL	CT	HCC	Surgery
Kang 2005 (22)	M,44	DLBL	PET-CT	Hepatitis	Liver biopsy
Nouwar 2018 (23)	F,55	DLBL	CT/MRI	Hepatitis	Liver biopsy
Lee 2013 (24)	F,57	DLBL	CT	Hepatitis	Liver biopsy
	M,59	PTCL	CT	Hepatitis	Liver biopsy
Li 2016 (25)	F,49	MALT	MRI	HCC	Surgery
Dong 2017 (26)	M,50	MALT	MRI	CC	Surgery
Xu 2021 (27)	F,63	MALT	MRI	HCC	Liver biopsy
Raimondo 2012 (28)	F	FL	US	Metastasis	Surgery
Chatelain 2006 (29)	F	MALT	CT	Metastasis	Surgery
Valladolid 2013 (30)	F,76	DLBL	CT	Hepatic Cyst	Surgery
Doi 2008 (31)	M,58	MALT	FDG-PET CT	HCC	Surgery

Male (M), female (F), diffuse large B-cell lymphoma (DLBL), mucosa-associated lymphoid tissue (MALT), peripheral T-cell lymphoma (PTCL), follicular lymphoma (FL), hepatocellular carcinoma (HCC), cholangiocellular carcinoma (CC), ultrasound (US), computed tomography (CT), magnetic resonance imaging (MRI), fluorodeoxyglucose-positron emission tomography examination integrated with computed tomography scanning (FDG-PET CT).

Notably, MALT lymphoma is a unique type of lymphoma that significantly differs from other indolent B-cell lymphomas. It is characterized by cellular heterogeneity of neoplastic cells. Under the microscope, reactive follicles are typically present, and the interfollicular space and marginal zone are both occupied by neoplastic cells. Occasionally, the follicles may contain an excess of marginal zone or monocytoid cells, giving them a neoplastic appearance, which is called follicular colonization (32). As typified by immunohistochemistry, MALT lymphoma cells express B-cell-associated antigens, such as CD20, CD22, CD19, CD79a, and CD79b. Conversely, neoplastic cells of MALT lymphomas seem to be negative for CD23, CD3, CD5, and CD10 (33). Usually, MALT lymphoma bears no relation to Bcl-1, Bcl-2, Bcl-3, or Bcl-6 rearrangements. Conversely, recent research indicated that the t(14;18)-(IgH;Bcl-2) translocation clusters sustain the role of HCV infection in the lymphoma development in HCV-positive individuals with gastrointestinal MALT lymphomas (34). In our present case, the immunohistochemistry results showed CD20+, CD79a+, CD43+, IgD+, and CD3−, CD5−, and CyClinD1−. The proliferation factor measured by Ki67 was ∼13%. Therefore, the diagnosis of MALT lymphoma was confirmed in this case by histopathologic assessment of tissue samples.

Routine HE staining can typically help establish the diagnosis of hepatocellular adenoma (HCA), but pathological variants are frequently observed. Based on recent discoveries on the genetics and molecular biology of HCA, hepatic adenomas can be divided into four subtypes: hepatocyte nuclear factor-1-alpha (HNF-1α)-mutated HCA (H-HCA), inflammatory HCA (I-HCA), β-catenin-mutated HCA (β-HCA), and unclassified HCA (U-HCA). More than 50% of all HCAs are I-HCAs, followed by H-HCAs, which make up 30%–40% of adenomas (18). Inflammatory infiltrates, sinusoidal dilatation, and thick-walled arteries are histological indicators of IHCA. H-HCA is distinguished by prominent steatosis, no cytological abnormalities, and the absence of inflammatory infiltrates (35). Evidently, the histopathological characteristics of hepatic lymphoma are quite different from those of hepatic adenoma.

The prognosis of PHL is not good because most patients have poor prognostic features, including constitutional symptoms, advanced age, elevated liver enzymes, and comorbid diseases (36). A study of 1,182 cases revealed marital status, age, and chemotherapy as independent prognostic factors for PHL (37). The median survival reported varies widely among the publications; according to a recent comprehensive analysis of 72 patients, the median survival of PHL is 15.3 months (38). A US population-based analysis of 1,372 patients with PHL reported that the most common NHL subtype was DLBCL (78.82%), followed by T/NK-cell lymphoma (4.69%), marginal zone lymphoma (MZL; 4.51%), Burkitt's lymphoma (4.33%), follicular lymphoma (3.13%), and small lymphatic lymphoma (2.49%), and the 1-, 3-, 5-, and 10-year overall survival (OS) rates for NHLs were 49.684%, 43.553%, 39.242%, and 28.364%, respectively (3). As for MALT lymphoma, both gastrointestinal MALT and non-gastrointestinal MALT have a relatively better prognosis, with 5-year OS rates higher than 90% and a 10-year OS rate of 75%–80%. Recurrences may occur several years after therapy, with a median recurrence period of 5 years, and these recurrences usually involve the same organ (60% of the cases) or other extranodal regions. Dissemination appears after a long disease-free interval in 30%–50% of patients; it typically affects adjacent mucosal locations with no involvement of peripheral blood or bone marrow (32). The 5-year OS rates of low-, low–intermediate-, high–intermediate-, and high-risk DLBCL patients are reportedly 73%, 51%, 43%, and 26%, respectively (39). An analysis of 1,712 patients with NHL, of which diffuse large B-cell lymphomas accounted for 61.3%, had revealed that the median time to progression and median overall survival of the whole group were 4.6 years and 8.4 years, respectively. Compared to B-NHL patients, T-cell lymphoma patients have a significantly shorter median survival rate (40).

The optimal therapy of PHL is still uncertain, and current treatment options include surgery, radiation therapy, chemotherapy, or a combination of these modalities (36). Surgical resection should be performed for patients with localized tumor to achieve better outcomes (41). It has been suggested that surgical operation followed by chemotherapy is a therapeutic approach for low-volume localized PHL (42). Nevertheless, combination chemotherapy should always be used for PHL because it is sensitive to chemotherapy and relapse after surgery is not uncommon. In addition, chemotherapy in combination is frequently recommended as the first line of treatment because it is the least invasive and increases overall survival. A retrospective analysis of 9 patients with PHL treated with liver resection revealed that postoperative chemotherapy offers a better outcome, and chemotherapy has been proven as the only prognostic factor for survival (9). As far as we know, CHOP (cyclophosphamide, doxorubicin, vincristine, and prednisone) is used to treat non-Hodgkin lymphoma, either with or without rituximab (R-CHOP), bendamustine, and lenalidomide (43). For patients with follicular NHL, several large-scale prospective randomized trials have established that incorporating rituximab into the first-line treatment would prolong remission (44). Presently, radiotherapy is the most diffusely studied treatment for patients with MALT lymphoma. Moderate-dose radiotherapy including perigastric lymph nodes and the stomach may provide good results with a high remission rate in gastrointestinal MALT patients (45). Mantle cell lymphoma is another uncommon aggressive B-cell lymphoma with poor prognosis and typically presents with advanced-stage disease and extranodal involvement with a predilection for bone marrow and gastrointestinal tract. The treatment of MCL is still a challenge and often leaves room for improvement; complete responses to standard chemotherapy regimens are uncommon (16).The therapeutic effect for MALT lymphomas is extremely heterogeneous, and thus, widely accepted treatment guidelines do not currently exist. In our case, the patient refused to undergo chemotherapy or radiotherapy. Considering the potential life-long risk of recurrence, long-term follow-up is still recommended for such patients.

## Conclusion

Primary hepatic lymphoma of MALT type is a rare, low-grade B-cell malignancy. Making an accurate preoperative diagnosis of this disease is usually difficult, and liver biopsy is an appropriate avenue to improve the diagnostic accuracy. After excluding common liver tumors, PHL might be suspected if liver lesions that do not involve other organs and present with elevated LDH and normal AFP levels are observed. In patients with a localized tumor lesion, hepatectomy followed by chemotherapy or radiotherapy should be considered to achieve better outcomes. Although this study describes an unusual type of hepatic lymphoma mimicking a benign tumor, it has its inherent limitations. More clinical studies are required to establish guidelines for the diagnosis and treatment of this rare disease.

## Data Availability

The raw data supporting the conclusions of this article will be made available by the authors, without undue reservation.

## References

[1] Padhan RK Das P Shalimar. Primary hepatic lymphoma. Trop Gastroenterol. (2015) 36:14–20. 10.7869/tg.239 26591949

[2] Dantas E Santos J Coelho M Sequeira C Santos I Cardoso C Primary hepatic lymphoma in a patient with cirrhosis: a case report. J Med Case Rep. (2020) 14:168. 10.1186/s13256-020-02471-0 32977834 PMC7519549

[3] Qiu MJ Fang XF Huang ZZ Li QT Wang MM Jiang X Prognosis of primary hepatic lymphoma: a US population-based analysis. Transl Oncol. (2021) 14:100931. 10.1016/j.tranon.2020.100931 33188980 PMC7672323

[4] Xing AY Dong XZ Zhu LQ Liu L Sun D Guo S. Clinicopathological characteristics and molecular phenotypes of primary hepatic lymphoma. Front Oncol. (2022) 12:906245. 10.3389/fonc.2022.906245 35832546 PMC9272565

[5] Cesaretti M Loustau M Robba C Senescende L Le Bian AZ. Reappraisal of primary hepatic lymphoma: is surgical resection underestimated? Crit Rev Oncol Hematol. (2018) 123:1–6. 10.1016/j.critrevonc.2018.01.004 29482772

[6] Salar A. Gastric MALT lymphoma and Helicobacter pylori. Med Clin (Barc). (2019) 152:65–71. 10.1016/j.medcli.2018.09.006 30424932

[7] Mehta N Jayapal L Goneppanavar M Ramakrishnaiah VPN. Primary hepatic lymphoma: a rare case report. JGH Open. (2019) 3:261–3. 10.1002/jgh3.12131 31276045 PMC6586568

[8] Eom D-W Huh JR Kang YK Lee YS Yu E. Clinicopathological features of eight Korean cases of primary hepatic lymphoma. Pathol. Int. (2004) 54:830–6. 10.1111/j.1440-1827.2004.01752.x 15533225

[9] Yang XW Tan WF Yu WL Shi S Wang Y Zhang YL Diagnosis and surgical treatment of primary hepatic lymphoma. World J. Gastroenterol. (2010) 16:6016–9. 10.3748/wjg.v16.i47.6016 21157979 PMC3007103

[10] Kawahara A Tsukada J Yamaguchi T Katsuragi T Higashi T. Reversible methotrexate-associated lymphoma of the liver in rheumatoid arthritis: a unique case of primary hepatic lymphoma. Biomark Res. (2015) 3:10. 10.1186/s40364-015-0035-2 25964853 PMC4426646

[11] Imrani K Znati K Amouri W Nassar I Billah NM. Primary hepatic lymphoma in liver cirrhosis: a rare case report. Radiol Case Rep. (2021) 16:2179–83. 10.1016/j.radcr.2021.05.028 34188736 PMC8218733

[12] Haque M Webber DL Kebarle P Shapiro RJ Yoshida EM. Hepatic lymphoma in a post renal transplant patient with chronic hepatitis B. Ann Hepatol. (2010) 9:91–2. 10.1016/S1665-2681(19)31686-2 20308728

[13] Muttillo EM Dégot T Canuet M Riou M Renaud-Picard B Hirschi S Primary hepatic lymphoma after lung transplantation: a report of 2 cases. Transplant Proc. (2021) 53:692–5. 10.1016/j.transproceed.2021.01.030 33531191

[14] Alves AMA Torres US Velloni FG Ribeiro BJ Tiferes DA D'Ippolito G. The many faces of primary and secondary hepatic lymphoma: imaging manifestations and diagnostic approach. Radiol Bras. (2019) 52:325–30. 10.1590/0100-3984.2018.0013 31656351 PMC6808615

[15] Ippolito D Porta M Maino C Pecorelli A Ragusi M Giandola T Diagnostic approach in hepatic lymphoma: radiological imaging findings and literature review. J Cancer Res Clin Oncol. (2020) 146:1545–58. 10.1007/s00432-020-03205-x 32296934 PMC11804346

[16] Albano D Laudicella R Ferro P Allocca M Abenavoli E Buschiazzo A The role of 18F-FDG PET/CT in staging and prognostication of mantle cell lymphoma: an Italian multicentric study. Cancers (Basel). (2019) 11(12):1831. 10.3390/cancers11121831 31769415 PMC6966583

[17] Szor DJ Ursoline M Herman P. Hepatic adenoma. Arq Bras Cir Dig. (2013) 26:219–22. 10.1590/s0102-67202013000300012 24190381

[18] Wong VK Fung AW Elsayes KM. Magnetic resonance imaging of hepatic adenoma subtypes. Clin Liver Dis (Hoboken). (2021) 17:113–8. 10.1002/cld.996 33868649 PMC8043715

[19] Kaneko K Nishie A Arima F Yoshida T Ono K Omagari J A case of diffuse-type primary hepatic lymphoma mimicking diffuse hepatocellular carcinoma. Ann Nucl Med. (2011) 25:303–7. 10.1007/s12149-010-0460-0 21234726

[20] Bohlok A De Grez T Bouazza F De Wind R El-Khoury M Repullo D Primary hepatic lymphoma mimicking a hepatocellular carcinoma in a cirrhotic patient: case report and systematic review of the literature. Case Rep Surg. (2018) 2018:9183717. 10.1155/2018/9183717 29850362 PMC5914115

[21] Lee J Park KS Kang MH Kim Y Son SM Choi H Primary hepatic peripheral T-cell lymphoma mimicking hepatocellular carcinoma: a case report. Ann Surg Treat Res. (2017) 93:110–4. 10.4174/astr.2017.93.2.110 28835888 PMC5566745

[22] Kang KM Chung WC Lee KM Hur SE Nah JM Kim GH A case of primary hepatic lymphoma mimicking hepatitis. Korean J Hepatol. (2005) 11:284–8. 16177555

[23] El Nouwar R El Murr T. Primary hepatic diffuse large B-cell lymphoma mimicking acute fulminant hepatitis: a case report and review of the literature. Eur J Case Rep Intern Med. (2018) 5:000878. 10.12890/2018_000878 30756046 PMC6346882

[24] Lee JA Jeong WK Min JH Kim J. Primary hepatic lymphoma mimicking acute hepatitis. Clin Mol Hepatol. (2013) 19:320–3. 10.3350/cmh.2013.19.3.320 24133672 PMC3796684

[25] Li LX Zhou ST Ji X Ren H Sun YL Zhang JB Misdiagnosis of primary hepatic marginal zone B cell lymphoma of mucosa-associated lymphoid tissue type, a case report. World J Surg Oncol. (2016) 14:69. 10.1186/s12957-016-0817-5 PMC4782304 26956381

[26] Dong S Chen L Chen Y Chen X. Primary hepatic extranodal marginal zone B-cell lymphoma of mucosa-associated lymphoid tissue type: a case report and literature review. Medicine (Baltimore). (2017) 96:e6305. 10.1097/MD.0000000000006305 28353562 PMC5380246

[27] Xu Z Pang C Sui J Gao Z. A case of primary hepatic extranodal marginal zone B-cell mucosa-associated lymphoid tissue (MALT) lymphoma treated by radiofrequency ablation (RFA), and a literature review. J Int Med Res. (2021) 49:300060521999539. 10.1177/0300060521999539 33730924 PMC8166399

[28] Raimondo L Ferrara I Stefano AD Cella CA D'Armiento FP Ciancia G Primary hepatic lymphoma in a patient with previous rectal adenocarcinoma: a case report and discussion of etiopathogenesis and diagnostic tools. Int J Hematol. (2012) 95:320–3. 10.1007/s12185-012-1025-x 22351247

[29] Chatelain D Maes C Yzet T Brevet M Bounicaud D Plachot JP Primary hepatic lymphoma of MALT-type: a tumor that can simulate a liver metastasis. Ann Chir. (2006) 131:121–4. 10.1016/j.anchir.2005.07.006 16246295

[30] Valladolid G Adams LL Weisenberg E Maker VK Maker AV. Primary hepatic lymphoma presenting as an isolated solitary hepatic cyst. J Clin Oncol. (2013) 31:e21–3. 10.1200/JCO.2012.44.9728 23169506

[31] Doi H Horiike N Hiraoka A Koizumi Y Yamamoto Y Hasebe A Primary hepatic marginal zone B cell lymphoma of mucosa-associated lymphoid tissue type: case report and review of the literature. Int J Hematol. (2008) 88:418–23. 10.1007/s12185-008-0153-9 18807227

[32] Raderer M Kiesewetter B Ferreri AJM. Clinicopathologic characteristics and treatment of marginal zone lymphoma of mucosa-associated lymphoid tissue (MALT lymphoma). CA Cancer J Clin. (2016) 66:153–71. 10.3322/caac.21330 26773441

[33] Isaacson PG Chott A Nakumura S. Extranodal marginal cell lymphoma of mucosa-associated tissue (MALT lymphoma). In: Swerdlow SH Campo E Harris NL, editors. WHO Classification of tumours of the haematopoietic and lymphoid tissues. Lyon, France: IARC Press (2008). p. 214–9.

[34] Libra M Gloghini A Malaponte G Gangemi P Re VD Cacopardo B Association of t(14;18) translocation with HCV infection in gastrointestinal MALT lymphomas. J Hepatol. (2008) 49:170–4. 10.1016/j.jhep.2008.03.031 18538438

[35] Bioulac-Sage P Balabaud C Zucman-Rossi J. Subtype classification of hepatocellular adenoma. Dig Surg. (2010) 27:39–45. 10.1159/000268406 20357450

[36] Noronha V Shafi NQ Obando JA Kummar S. Primary non-Hodgkin's Lymphoma of the liver. Crit Rev Oncol Hematol. (2005) 53:199–207. 10.1016/j.critrevonc.2004.10.010 15718146

[37] Zhang SL Chen C Rao QW Guo Z Wang X Wang ZM Incidence, prognostic factors and survival outcome in patients with primary hepatic lymphoma. Front Oncol. (2020) 10:750. 10.3389/fonc.2020.00750 32477954 PMC7239999

[38] Mastoraki A Stefanou MI Chatzoglou E Danias N Kyriazi M Arkadopoulos N Primary hepatic lymphoma: dilemmas in diagnostic approach and therapeutic management. Indian J Hematol Blood Transfus. (2014) 30:150–4. 10.1007/s12288-013-0263-2.21 25114399 PMC4115079

[39] Shi Q Shen R Wang CF Fan X Qian Y Ou-Yang BS Pretreatment liver injury predicts poor prognosis of DLBCL patients. Mediators Inflamm. (2017) 2017:7960907. 10.1155/2017/7960907 29109622 PMC5646333

[40] Trněný M Sálková J Dlouhá J Stříteský J. Hepatic involvement in patients with non-hodgkins lymphoma. Vnitr Lek. (2013) 59:606–11. 23909267

[41] Ugurluer G Miller RC Li YX Thariat J Ghadjar P Schick U Primary hepatic lymphoma: a retrospective, multicenter rare cancer network study. Rare Tumors. (2016) 8:6502. 10.4081/rt.2016.6502 27746888 PMC5064304

[42] Page RD Romaguera JE Osborne B Medeiros LJ Rodriguez J North L Primary hepatic lymphoma: favourable outcome after combination chemotherapy. Cancer. (2001) 92:2023–9. 10.1002/1097-0142(20011015)92:8<2023::aid-cncr1540>3.0.co;2-b 11596015

[43] Lewis WD Lilly S Jones KL. Lymphoma: diagnosis and treatment. Am Fam Physician. (2020) 101:34–41. 31894937

[44] Marcus R Hagenbeek A. The therapeutic use of rituximab in non-Hodgkin's Lymphoma. Eur J Haematol Suppl. (2007) 67:5–14. 10.1111/j.1600-0609.2006.00789.x 17206982

[45] Park HS Kim YJ Yang WI Suh CO Lee YC. Treatment outcome of localized Helicobacter pylori-negative low-grade gastric MALT lymphoma. World J Gastroenterol. (2010) 16:2158–62. 10.3748/wjg.v16.i17.2158 20440857 PMC2864842

